# ﻿*Carexyankouensis*, a new species of Cyperaceae from limestone landform in northern Guangdong, China

**DOI:** 10.3897/phytokeys.254.140929

**Published:** 2025-03-24

**Authors:** Ang Liu, Jian-jun Zhou, Lei Wu, Xun-lin Yu

**Affiliations:** 1 Central South University of Forestry & Technology, Changsha 410004, Hunan, China Central South University of Forestry & Technology Changsha China; 2 Hunan Agriculture and Forestry Industry Survey and Design Institute Co., Ltd, Changsha 410007, Hunan, China Hunan Agriculture and Forestry Industry Survey and Design Institute Co., Ltd Changsha China

**Keywords:** *Carex*, limestone landform, new species, taxonomy

## Abstract

*Carexyankouensis*, a new species of Cyperaceae (CarexsectionRhomboidales) from the limestone landform in northern Guangdong, China is described and illustrated. The new species is similar to *C.brevicuspis* C. B. Clarke, but differs in having shorter culms (10–15 cm vs 20–55 cm) and spikes (1–1.5 cm vs 3.7–7 cm), leaves wider (15–20–35 mm vs 5–10 mm) and lighter colored (pale green or yellow-green vs dark green), nutlet beak oblique (vs erect or slightly curved), and slightly thickened (vs thickened) style base. Following the IUCN Red List Criteria (IUCN 2024), *Carexyankouensis* is assessed as ‘Data Deficient (DD)’.

## ﻿Introduction

*Carex* L., belonging to Cyperaceae, encompasses approximately 2000 species which are distributed across all continents except Antarctica. Notably, the treatment in Flora of China features 527 of these species, with an impressive 260 being exclusive to China (Dai et al. 2010). Undoubtedly, *Carex* stands as one of the most diverse genera among seed plants worldwide, yet the intricacies of its classification pose significant challenges. Despite these difficulties, recent years have witnessed the publication of numerous novel *Carex* species for China (Li et al. 2022; Lu and Jin 2022; Lu et al. 2023; Li et al. 2024; Qiu et al. 2024) and other parts of the world.

In Flora of China (Dai et al. 2010), species of the genus *Carex* are classified into 42 sections and three subgenera. The treatment includes section Rhomboidales with 43 species. However, the taxonomic revisions of the section have not been entirely resolved yet. Jin and Zheng (2013) revised this section and recognized 40 species, along with six subspecies and four varieties, but due to the widespread distribution of the species of this section, new species may still exist in some special geomorphic areas, such as limestone regions. Indeed, some recently published species of this section, such as *C.duanensis* Z.C.Lu, Y.F.Lu & X.F.Jin (Lu et al. 2024), are distributed in such limestone landform areas.

In July 2021, during our investigation in the limestone area of northern Guangdong, we collected a distinct species of *Carex* which they grew on the walls of a karst cave. Although it neither bloomed nor bore fruit, the specimens were striking by their wide leaves, which reached a width of up to 35 mm, and the obvious small transverse veins between the leaf veins. We took two plants back to Changsha City for further observation and research. Fortunately, in November of that year, these two plants bloomed and provided us with mature nutlets for our research in March of the following year. At the same time, we also went to the corresponding phenological period to collect voucher specimens from the type locality. Through phenological observation and morphological research, we finally confirmed that this was a new species of C.sect.Rhomboidales.

## ﻿Material and methods

The specimens are mainly stored in the Herbarium of Forest Plants in Central South University of Forestry and Technology (CSFI). The morphological observation of the new species is based on field investigations, cultivated plants from the type locality, and specimen studies. Morphological research includes the length of rhizomes, the length, width and color of leaves, number of spikes, and the shape, size of bracts, glumes, utricles, and nutlets. We also use SEM to observe the nutlets which come from the holotype specimen we collected to ensure that the relevant descriptions were true. The sample preparation process and operating procedures of SEM refer to previous research by Lu et al. (2024). The conservation status of this new species is based on field observations in accordance with IUCN Red List guidelines (IUCN 2024).

## ﻿Taxonomic treatment

### ﻿ Carex yankouensis

Taxon classificationPlantaePoalesCyperaceae

X.L.Yu, A.Liu & J.J.Zhou sp. nov.

D9EBAE00-40D1-5F91-BC63-6624B3B96D0D

urn:lsid:ipni.org:names:77359144-1

Figs 1 , 2 , 3

#### Diagnosis.

This new species is similar to *C.brevicuspis* C. B. Clarke, but differs from it in having shorter culms (10–15 cm vs 20–55 cm) and spikes (1–1.5 cm vs 3.7–7 cm), leaves much wider (15–20–35 mm vs 5–10 mm) and lighter colored (pale green or yellow-green vs dark green), nutlet beak oblique (vs straight or slightly curved), and style base slightly thickened (vs conspicuously thickened) (Referring to Fig. 5, Table 1).

**Table 1.** Comparison of morphological characters between *Carexyankouensis* sp. nov. and *C.brevicuspis*.

Characters	*Carexyankouensis* sp. nov.	*C.brevicuspis*
Culms	10–15 cm high	20–55 cm high
Leaves	15–20(-35) mm wide	5–10 mm wide
	papery, soft	papery, hard
	pale green or yellow-green	dark green
	transverse veins between the leaf veins extremely distinct	transverse veins between the leaf veins diddly distinct
Spikes	terminal spike staminate, 1–3(-4) cm	terminal spike staminate, 2.5–4 cm
	lateral spikes 1–1.5 × 0.6–0.8 cm	lateral spikes 3.7–7 × 0.9–1 cm
Nutlets	beak oblique	beak straight or slightly curved
	style base slightly thickened	style base conspicuously thickened

**Figure 1.**
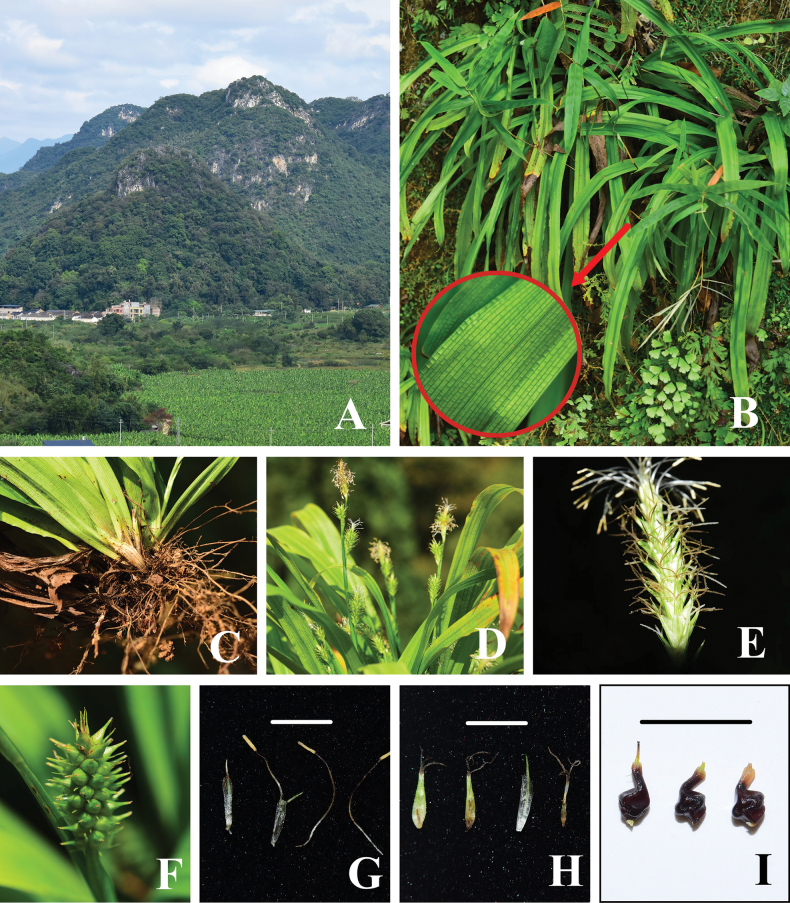
*Carexyankouensis* sp. nov. **A** habit **B** plants, transverse veins between the leaf veins extremely distinct in the red circle **C** rhizome **D** inflorescences **E** spikelet in flower **F** spikelet in fruit **G** female glumes and stamens **H** yong utricles, Male glume and stigmas **I** nutlets. Photographs by Ang Liu. Scale bars: 5 mm.

**Figure 2.**
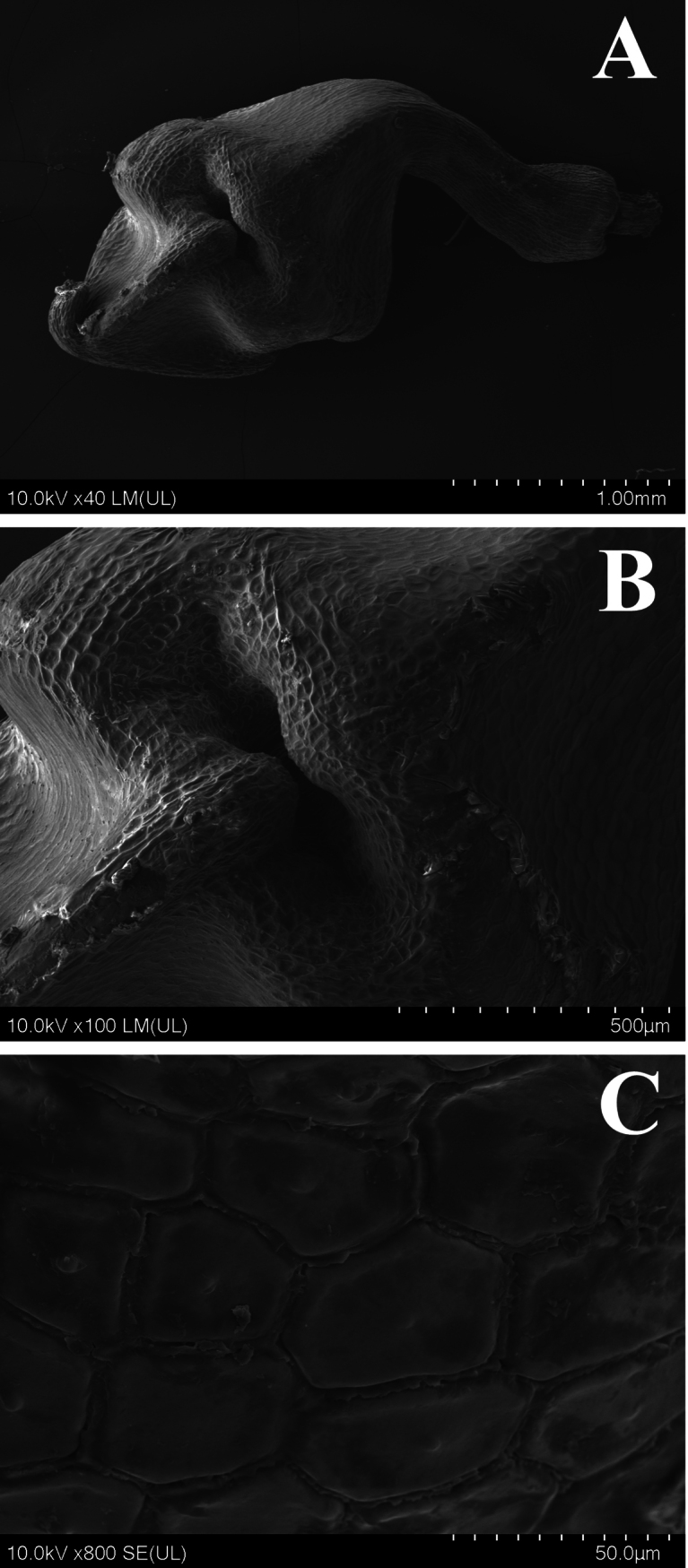
SEM micromorphology of nutlets of *Carexyankouensis* sp. nov. **A** overview **B** angle constricted at middle **C** sexine ornamentation. The nutlets are from the holotype: *Ang Liu* LAYD01, CSFI 076290. Scale bars: 1 mm (**A**); 500 μm (**B**); 50 μm (**C**).

**Figure 3.**
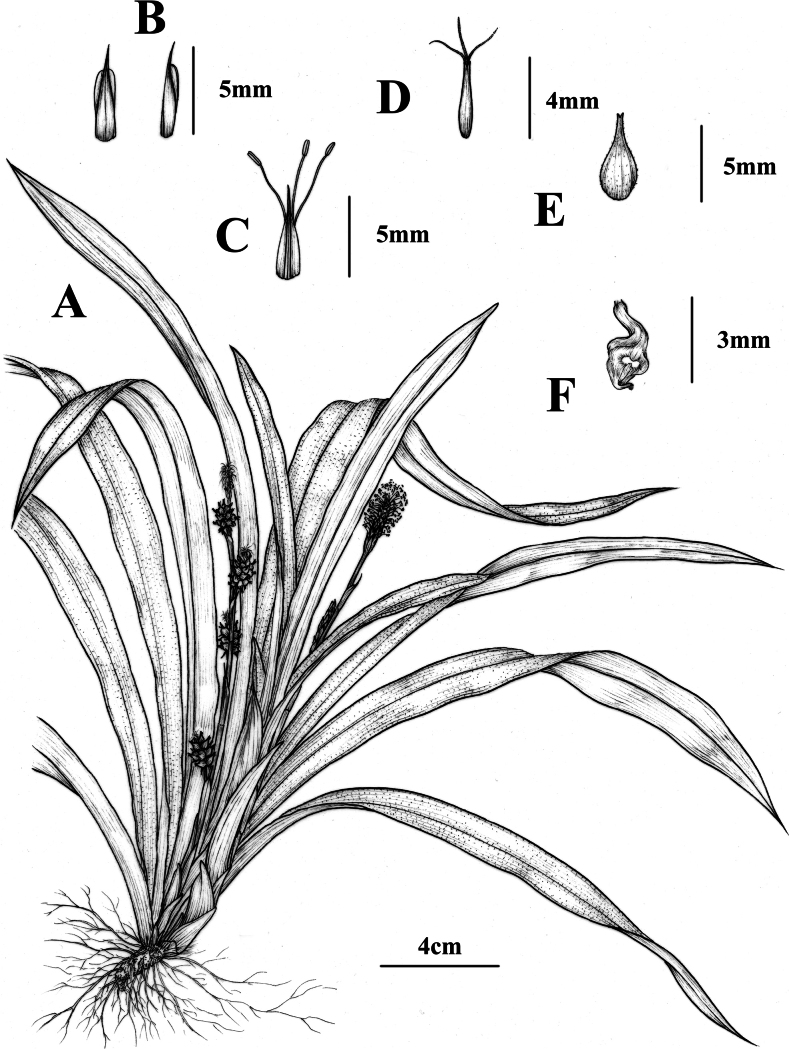
*Carexyankouensis* sp. nov. **A** plant **B** glumes **C** female glume and stamens **D** young utricle and stigmas **E** utricle **F** nutlet. Drawn by PhD Jing Tian; based on the holotype: *Ang Liu* LAYD01, CSFI 076290 and cultivated plants from type locality.

#### Type.

**Figure 4.**
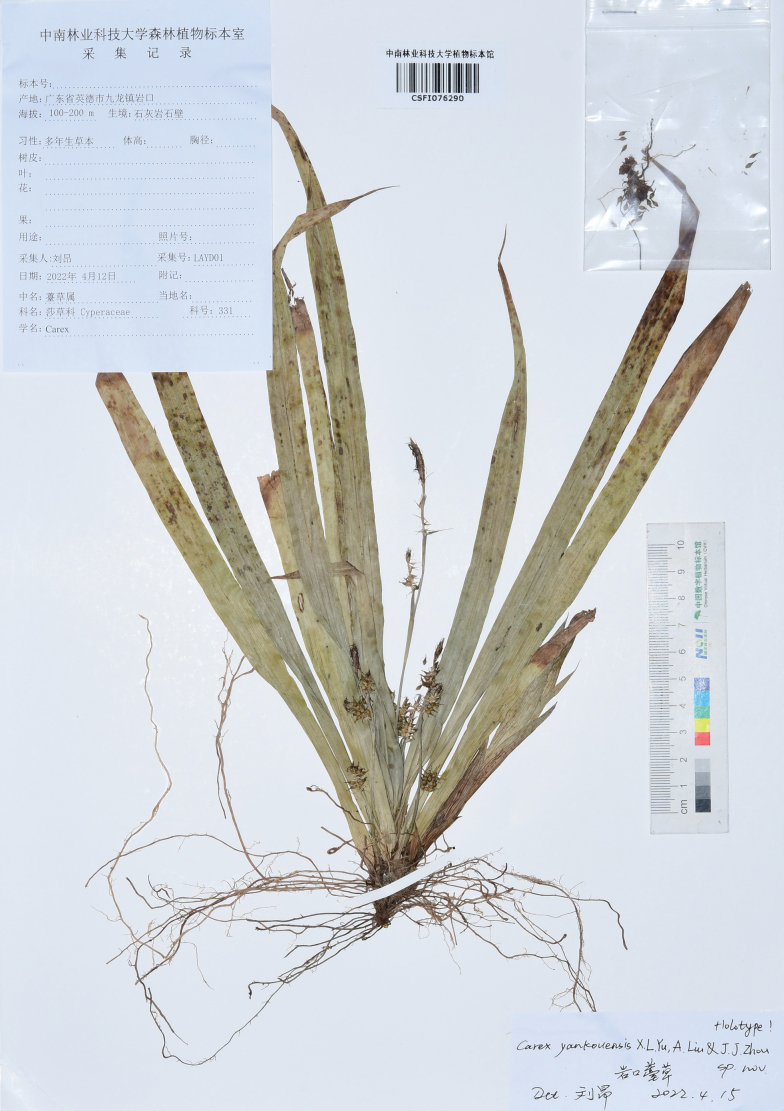
Holotype of *Carexyankouensis* sp.nov. (*Ang Liu* LAYD01, CSFI 076290).

China • Guangdong: Qingyuan City, Yingde County, Jiulong Town, Yankou, in dry limestone, elevation ca. 100–200 m, 4 April 2022, *Ang Liu* LAYD01 (Holotype CSFI!, isotype HIB!, CSH! & ZJFC!) (Referring to Fig. 4).

**Figure 5.**
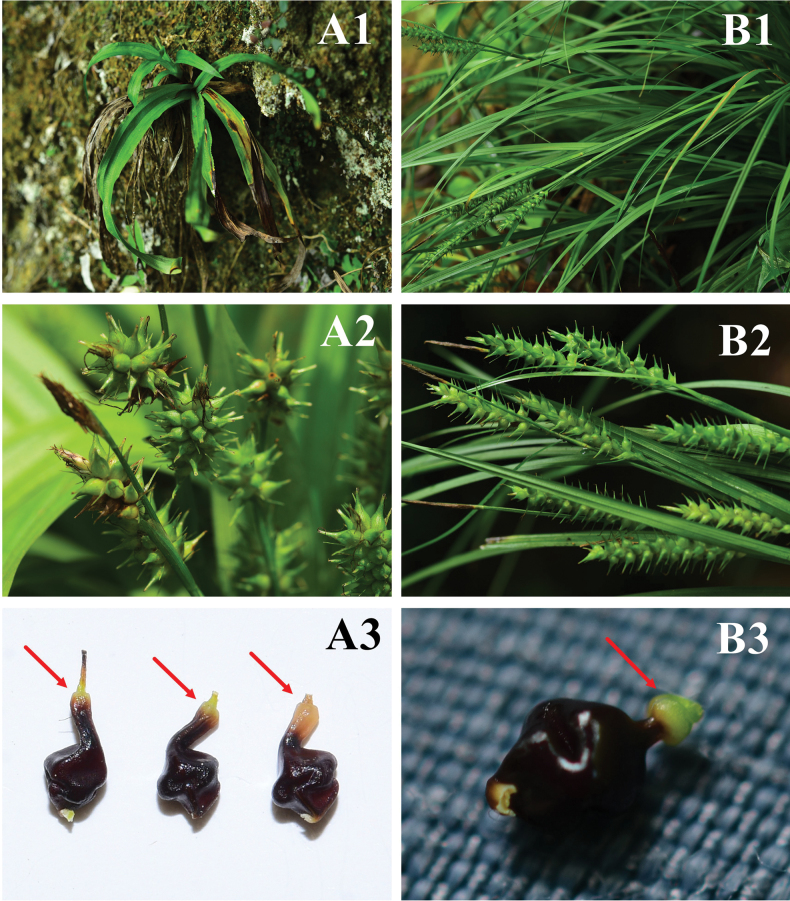
Morphological comparison of *Carexyankouensis* sp. nov. (**A1–A3**) and *C.brevicuspis* (**B1–B3**) **A1, B1** plants **A2, B2** infructescence **A3, B3** nutlets. Photographs by Ang Liu.

#### Description.

Perennial herbs. ***Rhizome*** short, stout. ***Culms*** 10–15 cm tall, blunt trigonous, smooth, base clothed with brown fibrous sheaths. ***Leaves*** up to 30 cm long and 15–20(-35) mm wide, longer than culms, blades papery, soft, broadly linear, pale green or yellow-green, flat, margin entire, apex acuminate or tailed, distinctly transverse veins between the leaf veins. ***Bracts*** leaflike, much shorter than inflorescence, sheathing. ***Spikes*** 4–5, distant, the proximal spike usually nearly basal and far from the distal ones; terminal spike staminate, 1–3(-4) cm, linear, with a peduncle ca. 3 cm; lateral spikes mostly pistillate, sometimes with several male flowers at apex, 1–1.5 × 0.6–0.8 cm, narrowly cylindric, densely flowered, the proximal-most one with a peduncle 3–5 cm, smooth. ***Glumes*** ca. 3 × 1 mm, staminate and pistillate ones similar in morphology, linear-lanceolate, pale, edges transparent, green 3-veined costa ending at apex, the tip rounded. ***Utricles*** ca. 5 × 2 mm (including beak), longer than or nearly equaling glumes, obliquely patent, the body ovoid or obovoid, pale green, the walls herbaceous, the surface sparsely pubescent, many veined, contracted at both ends, the apex abruptly contracted into a ca. 3 mm long beak, orifice 2-lobed with sharp teeth. ***Nutlets*** ca. 2 × 1.5 mm, black-purple, ovate, trigonous, with 3 angles constricted at middle, faces concave at base, the epidermic cells forming an ornamentation of irregular polygons, base curved stipitate, apex abruptly contracted into an oblique beak, beak ca. 1 mm, slightly annulate at orifice; style base slightly thickened; stigmas 3.

#### Phenology.

Flowers observed from November to December, fruits from March to April.

#### Etymology.

The epithet of this new species is derived from the type locality. ‘Yankou’ is the locality name, which means the entrance of a karst cave in Chinese.

#### Distribution and habitat.

This new species is currently only found in the limestone landform areas of Jiulong Town, and usually grows on the walls of limestone.

#### Additional specimens examined

**(Paratypes).** China • Guangdong: Qingyuan City, Yingde County, Jiulong Town, Hui long Park, in dry limestone, elevation ca. 120 m, 9 November 2023, *Ang Liu* LAYD06 (CSFI!).

#### Conservation status.

At present, we have only found two populations with a total of about 200 individuals in the limestone areas of Jiulong Town. However, there are vast limestone landforms near the type location, and there may be distribution of this new species in these areas. Of course, we need a broader and deeper investigation to confirm that. According to the IUCN red list criteria (IUCN 2024), the conservation status of the new species should be better categorized as ‘Data Deficient (DD)’.

## Supplementary Material

XML Treatment for Carex yankouensis

## ﻿Additional information

### Author ORCIDs

Ang Liu https://orcid.org/0000-0001-6281-7145

### Data availability

All of the data that support the findings of this study are available in the main text.

## References

[B1] Dai LK Liang SY Zhang SR Tang YC Tetsuo K Gordon CT (2010) *Carex*. In: Wu ZY Raven PH (Eds) Flora of China, Vol.23. Science Press, Beijing & Missouri Botanical Garden Press, St. Louis, Beijing, 285–461. https://www.iplant.cn/info/Carex?t=foc

[B2] IUCN (2024) Guidelines for Using the IUCN Red List Categories and Criteria. version 16. Prepared by the Standards and Petitions Subcommittee of the IUCN Species Survival Commission. http://www.iucnredlist.org/resources/redlistguidelines

[B3] Jin XF Zheng CZ (2013) Taxonomy of CarexsectionRhomboidales (Cyperaceae). Science Press, Beijing, 1–237.

[B4] Li YY Chen YP Jiang LQ Liu ED Luo Y Peng H (2022) ﻿*Carexmalipoensis* (Cyperaceae), a new species from southeast Yunnan, China. PhytoKeys 188: 19–29 10.3897/phytokeys.188.75401 35095290 PMC8760235

[B5] Li YL Deng SW Luo JC Li MX Zou LT Zeng QG Chen HF (2024) *Carexqingyuanensis* (Cyperaceae), a new species from Guangdong, China, PhytoKeys 241: 155–168. 10.3897/phytokeys.241.117734 PMC11066503 38706583

[B6] Lu YF Jin XF (2022) Notes on *Carex* (Cyperaceae) from China (VIII): five new species and a new variety from southern and south-western China. PhytoKeys 188: 31–47. 10.3897/phytokeys.188.77776 35095291 PMC8760236

[B7] Lu YF Lu ZC Duan YH Zhang K Jin XF (2023) ﻿Notes on *Carex* (Cyperaceae) from China (IX): three new species of section Mitratae s.l. PhytoKeys 225: 153–164. 10.3897/phytokeys.225.101410 PMC10194776 37213819

[B8] Lu ZC Lu YF Chang SL Mo ML Jin XF (2024) ﻿*Carexduanensis* (Carexsect.Rhomboidales), a new species of Cyperaceae from limestone areas of Guangxi, China. PhytoKeys 241: 221–228. 10.3897/phytokeys.241.121098 38737292 PMC11082484

[B9] Qiu XD Lu YF Jin XF (2024) ﻿*Carexlinanensis* (sect. ﻿*Mitratae*), a new species of Cyperaceae from Zhejiang, East China. PhytoKeys 241: 121–130 10.3897/phytokeys.241.119176 38665212 PMC11043649

